# Mr. Chevalier's Case of Supposed Rabies

**Published:** 1810-03

**Authors:** T. Chevalier

**Affiliations:** South Audley Street


### To Dr. ADAMS.

Dear Sir,

An erroneous opinion having been entertained in the neighbourhood where the following Case occurred, that the patient died of Hydrophobia, I think it may be proper to insert in your Journal the account Mr. Wakefield, who attended her, has sent me of the disease, and to subjoin the appearances on dissection.

Sir,

"Sir,

"ON the 16th of December, Ann Fidler, No. 14, Union Place, Battle Bridge, received a lacerated wound on the face from the bite of a large dog, which we supposed was not mad, from his remaining tearing the child, until a man with difficulty drove him away. The wound healed kindly, and she remained perfectly well until the 2d of February, when she had an attack of fever, attended with sickness at the stomach, which an emetic removed. On the following days, that is, to the 6th, the fever continued with but little sickness, but attended with general pain in the limbs and head, for which she was taking saline and antimonial medicines. On the 6th she complained of violent pain, attended with sickness. I applied a blister, and on the 7th found the head much relieved; but a sudden difficulty of swallowing came on, insomuch that every thing she attempted to swallow, instantly returned by the nostrils, but unattended with any dread at the sight of liquids. In the evening her existence was suddenly closed, as if by suffocation. There was delirium at intervals during the whole of her illness.

"I am, Sir,

"Your obedient servant,

"THOMAS WAKEFIELD."

---

I saw this girl, at the desire of her parents, in the afternoon of Wednesday the 7th of February, which was the last day of her illness. There was then no appearance which indicated the approach of dissolution. She was sensible; her pulse about 120, regular, and tolerably strong. I desired her to drink some liquid which was in a cup by the bed. She took it into her mouth, and endeavoured to swallow it, but it was instantly thrown back with a considerable quantity of mucus. The attempt to swallow was however made without any repugnance, and the failure of that attempt evidently arose only from absolute inability to exert the muscles of deglutition. I examined the throat, and found the left tonsil, and the arch of the palate on that side, a good deal enlarged; but not sufficiently to explain the difficulty or rather impossibility of swallowing, which her mother distinctly stated had only come on late on the preceding day.

I was much surprised when I was informed she had died suddenly that very evening, especially as there had not been the smallest difficulty of respiration. The parents and nd myself were equally desirous that the cause of her death should be ascertained by dissection; I therefore examined the body on Friday the 9th instant, in the presence of Mr. Wakefield and several other medical gentlemen.

I first of all took out the larynx, pharynx, and tongue, *en masse*, in order to ascertain the state they were in, with as little disturbance as possible. The posterior half of the tongue was swollen, and the fungiform papillæ appeared in consequence much larger than usual. The glottis was closed completely, and the larynx filled, by a quantity of viscid, glairy mucus, which it was rather difficult to detach, and which had evidently been the immediate cause of her death, by falling down and producing suffocation, for there was no mucus whatever found in the trachea below the larynx, or in its branches, nor was the inside of the larynx itself inflamed. The pharynx showed slight marks of inflammation; and on laying open the œsophagus, the inflammation appeared to have increased in regular gradation from its upper part to its termination in the cardia, where it was coated by a layer of coagulable lymph; and from about an inch above its termination, at the posterior part, was completely gangrenous, and tore with the slightest force. The appearance of inflammation extended into the stomach for about two inches round the cardia, chiefly however on the posterior part, in a continued line from the gangrened œsophagus.

The stomach contained a considerable quantity of acrid matter, tinged with bile. I never recollect to have seen a stomach so generally eroded by the gastric juice in so short a period after death.

The brain was in every respect healthy; and no fluid whatever effused, either between its membranes or within the ventricles.

There was something peculiar in the effluvia from this body, which though not offensive from putrefaction, yet gave me and one of my pupils a severe head-ach, which continued till the next day.

I am, &c.

T. CHEVALIER.

